# Design and Implementation of Miniature Multi-Mode 4 × 4 MIMO Antenna for WiFi 7 Applications

**DOI:** 10.3390/mi16121331

**Published:** 2025-11-26

**Authors:** Weizhen Lin, Kaiwen Du, Xueyun Jiang, Yongshun Wang

**Affiliations:** 1 National Key Laboratory of Microwave Imaging, Aerospace Information Research Institute, Chinese Academy of Sciences, Beijing 100094, China; linweizhen23@mails.ucas.ac.cn; 2 School of Electronics, Electrical and Communication Engineering, University of Chinese Academy of Sciences, Beijing 100049, China; 3 School of Electronic and Information Engineering, Lanzhou Jiaotong University, Lanzhou 730070, China; 11200682@stu.lzjtu.edu.cn; 4 School of Information Science and Engineering, Lanzhou Bowen College of Science and Technology, Lanzhou 730101, China; 13993102058@139.com

**Keywords:** WiFi 7, MIMO antenna, wideband, miniaturization, mutual coupling reduction

## Abstract

The compact and wideband patch antennas applied to WiFi 7 multiple-input multiple-output (MIMO) antenna systems are presented. The MIMO antenna structure consists of four multi-branch radiating patches fed by coupled microstrip lines, which occupies a size of $32\times 32\times 1\;{mm}^{3}$. By exploiting multiple resonant modes, an impedance bandwidth of 37% (5.07–7.37 GHz) achieves a reflection coefficient of less than −10 dB and fully encompasses both WiFi 7 high-frequency ranges. To alleviate mutual coupling, two decoupling structures, named complementary split-ring resonators (CSRRs), are employed between the MIMO elements to interact with the undesirable surface current; furthermore, the proposed orthogonal placement of four elements further minimizes radiation coupling. Consequently, the proposed array achieves measured isolations greater than 14.5 dB and 11 dB at 5 GHz and 6 GHz bands, respectively. The prototype of the proposed MIMO antenna has been manufactured. It has also been measured and the results show similarity with the simulations. The measured radiation pattern and the diversity performance, including the envelope correlation coefficient (ECC), diversity gain (DG), and multiplexing efficiency, are calculated, and they verify the outstanding diversity characteristics of the proposed MIMO antenna. This makes it a promising solution for emerging WiFi 7 wideband applications.

## 1. Introduction

Recently, an increasing number of mobile terminals require high-quality communication services, including higher data acquisition capacity, faster transmission speed, and enhanced reliability [1]. To meet these requirements, signal transceiver technologies have evolved from the early single transmission technique to the multiple-input multiple-output (MIMO) technique [2]. And the crucial components in this progress are antennas, whose performance directly determines the communication quality. Recent developments in high-frequency and miniaturized components, such as terahertz rectifiers [3], further illustrate the broader technological trend toward compact, broad-band wireless systems, complementing the design strategies employed in modern MIMO antennas. Nevertheless, two major challenges remain in designing MIMO antennas for such compact devices: achieving sufficient isolation between antenna elements and maintaining high efficiency within limited form factors.

Firstly, it is crucial for MIMO antennas to maintain a compact and portable form factor to enhance user convenience. Therefore, it takes one challenge for MIMO antennas, which is miniaturization, and overcomes the mutual coupling effect [4]. Various techniques have been employed for antenna miniaturization. One approach is to modify the antenna geometry to extend the effective current path length, thereby lowering the resonant frequency without enlarging the structure [5]. Another approach involves loading the antenna with materials that possess a high refractive index [6,7]. The reasons that lead to mutual coupling effects are radiation coupling between adjacent elements in free space and surface current coupling in the shared ground plane, which degrades the signal independence of the MIMO ports [8]. Decoupling structures typically involve adding additional components between adjacent elements and can be further divided according to their coupling suppression mechanisms. One method introduces new electromagnetic propagation paths to counteract the old coupling, which can be found in neutral lines [9,10,11], parasitic decoupling patches [12], decoupling networks [13,14], and so on. Another method is to control the surface current distribution by employing defect grounding structures [15], grounding branches [16], electromagnetic band-gap structures [17,18], and so on. In contrast, self-decoupling MIMO antennas achieve isolation by exploiting the orthogonality of their inherent modes [19,20,21], effectively suppressing mutual coupling without the need for extra decoupling elements.

Secondly, to solve channel congestion, narrow bandwidth, and high latency in mobile terminals, new bandwidth demands have also emerged. And it provides MIMO antennas with another challenge: wideband design. For wireless routers, traditional WiFi bands work on 2.4 GHz. Recently, WiFi 7 under IEEE 802.11be standard has been officially finalized. Although WiFi 7 operates in the same three frequency bands as WiFi 6/6E (2.4 GHz, 5 GHz, and 6 GHz), it supports channel bandwidths of 320 MHz which is an enhancement compared to 160 MHz of WiFi 6. This wider bandwidth significantly increases the requirements on antenna impedance bandwidth and radiation stability. To enhance antenna bandwidth, several effective techniques have been proposed, such as employing impedance matching networks [22], exciting multiple resonant modes [23,24], and introducing parasitic patches [25,26,27].

Several studies have investigated MIMO antenna designs for WiFi applications. In [28], a multi-frequency MIMO antenna integrating a cosine-shaped monopole, split-ring resonators (SRRs), and Archimedean spiral meta-surfaces works on the WiFi 6E high-band. The design achieves tri-band operation at 3.5, 5.8, and 6.9 GHz and an isolation improvement of over 30 dB, while average efficiency is about 89%. However, the excellent performance of this design relies on a complex decoupling structure. Furthermore, to achieve effective decoupling a certain height must be maintained between the meta-surface and the antenna, approximately half a wavelength at 5 GHz, which increases the profile height of the MIMO antenna and reduces the integration. Research in [29] proposes an eight-port MIMO antenna with a configuration of 12,730 ${mm}^{3}$. The antennas are microstrip patch antennas and employ feed coupling through multiple microstrip line branches on a single-layer substrate. A decoupling structure is not adopted, and ensuring isolation relies on an orthogonal layout. The MIMO elements isolation exceeds 10 dB at frequencies of 0.85–1.10 GHz, 1.71–2.83 GHz, and 4.47–7.40 GHz. It should be noted that the mentioned multi-branch coupling to achieve multi-band operation introduces various resonant paths, where each branch may excite its own mode. As a result, the field distribution is different across different bands, causing in-band radiation pattern instability. For a MIMO system, such pattern instability may lead to coverage blind spots. A three-port MIMO antenna is designed in [30]. A step-impedance resonator (SIR) meta-surface is employed in the design to achieve miniaturization. The proposed antenna achieves a wide bandwidth of 4.68–5.75 GHz while maintaining isolation better than 25 dB, which means it is ideal for 5G wireless routers. SIR meta-surface units are sensitive to geometric dimensions. They impose stringent requirements on manufacturing tolerances and PCB alignment, which can significantly affect resonant frequencies and matching performance. In [31], an eight-port MIMO antenna is proposed. The broadband characteristics of this antenna are achieved through the parallel operation of slot antennas and patch antennas operating at two frequencies. The antenna’s frequency range is 2.6 GHz to 7.46 GHz with an isolation better than 12 dB, making it suitable for WiFi 6E/7 applications. However, the spacing between antenna elements is approximately half a wavelength, indicating potential for further miniaturization. And in [32], a 4-port MIMO applied for wireless routers is designed. The bandwidth is about 13%. That is between 5.13 and 5.80 GHz. And the isolation is above 21 dB. The antenna radiation unit is designed on a dielectric substrate perpendicular to the floor, leaving potential for further optimization in terms of integration. In this article, we explore the possibility of arranging MIMO antennas on one dielectric substrate and reducing the size to improve integration further. This will bring about more serious mutual coupling problems, and the solution to address these is also provided.

In this article, a 4 × 4 MIMO antenna with compact and wideband characteristics composed of four patch elements is proposed for wireless router applications. Each antenna element adopts a multi-branch radiating patch and a coupled-feed microstrip line. Owing to the multi-mode resonance in the proposed structure, the impedance bandwidth reaches 37%, which ranges from 5.07 to 7.37 GHz, fitting WiFi 7 high-bands. Without increasing MIMO antenna size and profile height, dual CSRRs are added on the corners of the ground plane between adjacent elements to reduce coupling currents. By confining the coupling surface currents within the CSRRs and employing orthogonal radiation configurations, the proposed MIMO antenna maintains simulated isolations over 14.5 dB and 11 dB during the 5 GHz and 6 GHz bands of WiFi 7, respectively. The fabricated MIMO antenna prototype is tested. The measurement has good agreement with simulations. The measured radiation pattern exhibits omnidirectional characteristics, and the total efficiency ranges from 50% to 79% and from 45% to 80% in the 5G and 6 GHz bands, respectively. Furthermore, calculated ECCs remain below 0.16 and 0.2 in the 5 GHz and 6 GHz bands, respectively. Diversity gain and multiplexing efficiency are also calculated to discuss MIMO diversity characteristics further. The architecture of the following sections is organized as below: Section 2 describes the configuration and characteristics of the proposed MIMO antenna, including its antenna structure and the working principle, with detailed analyses of its wideband characteristics, decoupling behavior, and parametric performance. Section 3 presents the implementation and performance of the 4 × 4 MIMO antenna prototype. S-parameter characteristics, radiation pattern, total efficiency, and peak gain is measured. And its diversity performance is discussed in Section 3.2. Finally, Section 4 concludes the paper and outlines potential directions for future work.

## 2. Configuration and Characteristics of Proposed MIMO

### 2.1. MIMO Antenna Structure

**Figure 1:**
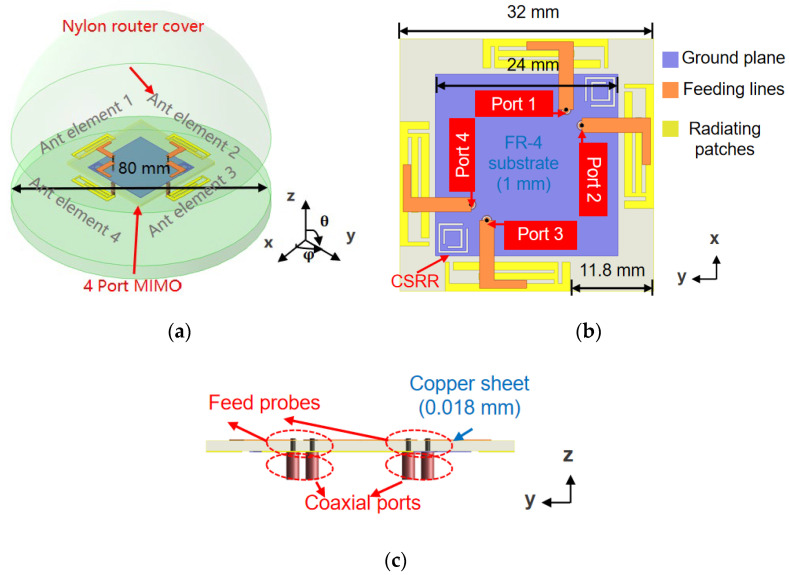
Geometry of proposed four-port MIMO antenna. (**a**) Main perspective; (**b**) top perspective; (**c**) side perspective.

Figure 1a depicts a four-port MIMO antenna configuration, which is designed for next-generation wireless routers. The antenna was optimized using ANSYS HFSS software 2023 to achieve wideband coverage across the new WiFi 7 frequency ranges of 5.150–5.835 GHz and 5.925–7.125 GHz. The antenna adopts a planar microstrip configuration and the substrate is FR-4 dielectric ($\varepsilon_{r}=4.25$ and tan δ=0.018) with a size of $32\times 32\times 1\;{mm}^{3}$, with two clearance areas of $11.8\times 4\;{mm}^{2}$, as shown in Figure 1b, which can be utilized for integrating traditional 2.4 GHz WiFi bands in future designs. Among them, elements 1 and 2, as well as elements 3 and 4, are placed adjacent to each other, forming two orthogonally arranged antenna pairs. Figure 1c is the antenna feed structure and each microstrip line is fed by copper probes and 50 Ω coaxial ports directly.

**Figure 2:**
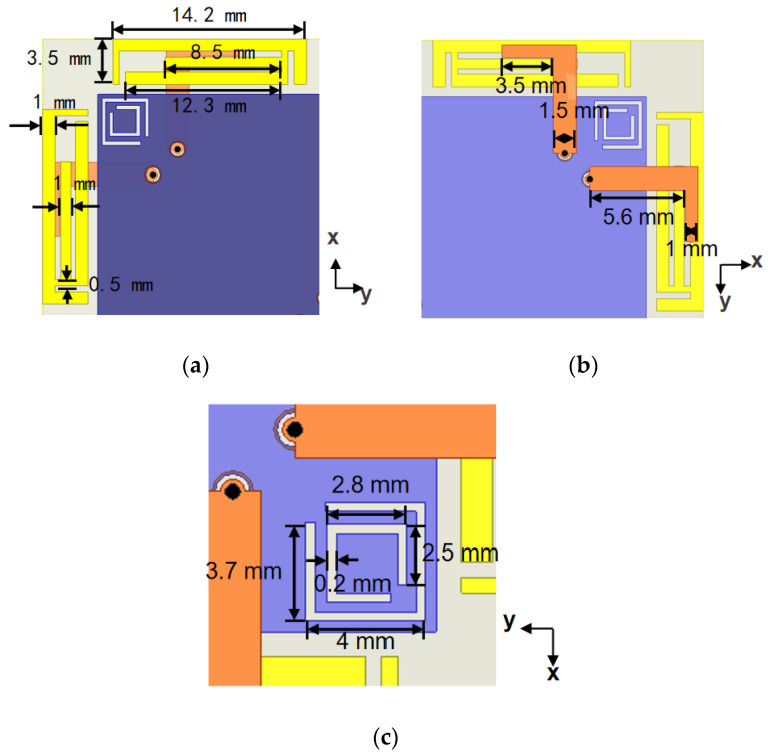
Structure and parameters of aerial elements. (**a**) Details of the radiating patches; (**b**) details of the feed lines; (**c**) details of the CSRR structures.

Figure 2 shows the detailed structure and parameters further. In Figure 2a, each antenna element has three main parts: a bent feed line printed on the front side of 1 mm thickness substrate, a copper sheet substrate back serving as a ground plane, and a multi-stub microstrip patch printed on the same side as the ground plane. To overcome the bandwidth limitation of conventional microstrip antennas, each element is excited through an L-shaped microstrip line and the radiating patch electromagnetic coupling. The feed line short end is linked to the inner probe of the coaxial port via a pad, while the copper ground plane is attached to the coaxial port’s outer conductor. Due to this coupling configuration introducing additional distributed capacitance, the resonance band is enhanced and the total size is reduced, which is an essential feature for compact antenna integration. Figure 2b depicts the microstrip feed line, which consists of two strip sections with different widths. This configuration enables impedance variation and matches the coupling mode impedance in a wideband. Because of the compact layout of the proposed design and the elements sharing a common ground plane, surface current coupling between adjacent elements is inevitable. To mitigate this undesired coupling, two CSRR structures are introduced between each pair of adjacent elements on the reference ground, as depicted in Figure 2c. The introduction of these CSRRs effectively improves the MIMO element isolation while exciting additional resonant modes on the ground plane, thereby further extending the proposed structure bandwidth.

### 2.2. Working Principle

#### 2.2.1. Analysis of the Wideband Characteristics

**Figure 3:**
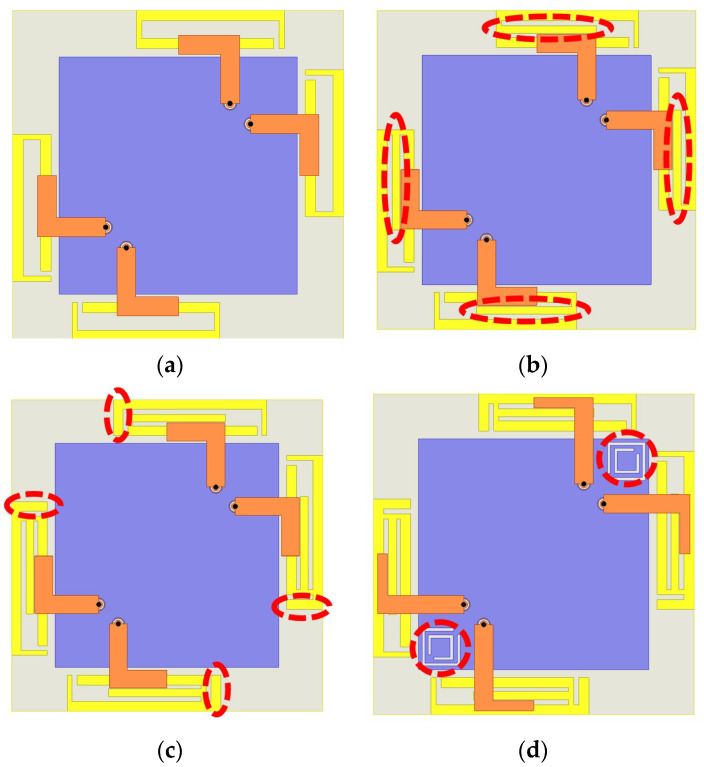
Changes in structure. (**a**) Model 1; (**b**) Model 2; (**c**) Model 3; (**d**) proposed model (Model 4).

**Figure 4:**
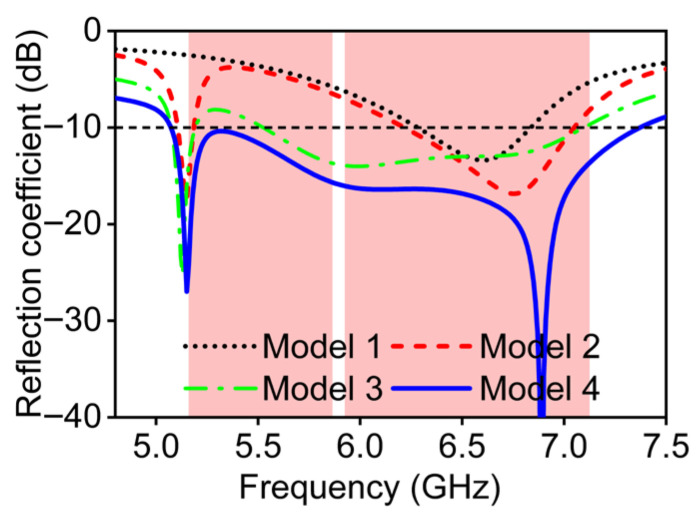
Reflection coefficient of four simulated evolution models (Model 1–4).

The multi-mode resonance principle is adopted to explain wideband characteristics. Before that, to clearly illustrate the operating mechanism, Figure 3 presents the structural evolution of the antenna from Model 1 to Model 4, while the bandwidth variations in these four models are shown by Figure 4. The red circle in Figure 3 represents the optimized structure during the antenna evolution process. The red area in Figure 4 represents the target frequency band of the antenna design, while the dotted lines represent the reference line with a reflection coefficient of -10 dB. This section reveals the relationship between the resonance contribution and the corresponding structural modifications, and identifies the contribution of each radiating part to the resonant frequencies through simulated surface current distributions. The red shade regions, from 5.150 to 5.835 GHz and 5.925 to 7.125 GHz, in Figure 4 denote the two operating bands of the WiFi 7 systems, which also represent the targeted frequency bands of this antenna design.

As shown in Figure 3a, the structure originates from a rectangular ring branch whose total length is approximately one wavelength at 6.6 GHz on the FR-4 substrate. The feed line positioned on the substrate’s front surface couples through the FR-4 material to excite one resonant mode of the antenna. As observed in Figure 4, this mode occurs around 6.6 GHz.

To achieve a wider bandwidth, it is essential to generate multiple resonant modes. Therefore, as illustrated in Figure 3b, a new branch is introduced at the center of the rectangular ring to influence the distribution of the resonance currents. The simulated reflection coefficient indicates that this configuration effectively excites two resonant modes, which are a lower-frequency resonance at 5.1 GHz and a higher-order mode shifted from 6.5 GHz to 6.7 GHz. Although this structure exhibits wideband potential suitable for WiFi 7 applications, the mid-frequency still does not exhibit satisfactory impedance matching.

**Figure 5:**
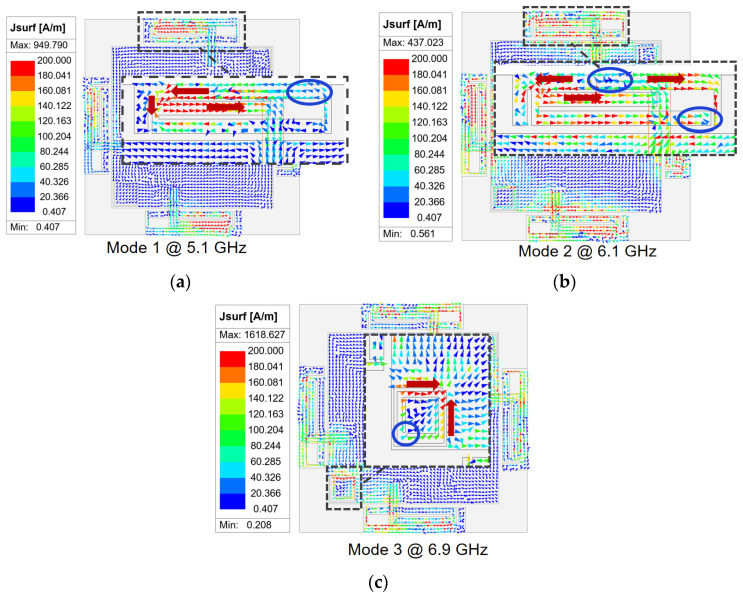
Simulated distributed current of the antenna surface for each resonant modes. (**a**) 5.1 GHz; (**b**) 6.1 GHz; (**c**) 6.9 GHz.

Figure 3c Model 3 tries to introduce an open-end branch at the bent side of the rectangular ring in an attempt to optimize impedance performance, which leads to new resonance behavior. The added branch increases the distributed capacitance and the resonant frequency becomes lower than before. For Model 3, the reflection coefficient from 5.5 GHz to 7.1 GHz is below −10 dB, covering part of the WiFi 7 high-band. Model 4 introduces dual CSRRs on the copper plane which serve as reference ground between adjacent antenna elements, as shown in Figure 3d. According to the CSRRs and as observed in the reflection coefficient results of Model 4, three distinct resonant modes appear at 5.1, 6.1, and 6.9 GHz, respectively. To better explore the multi-mode resonance principle, Figure 5 provides the corresponding current distribution simulated results at the above three resonant frequencies, where blue circles represent wave nodes in the current distribution, arrows indicate the direction of current flow, and the black dotted box highlights the focused antenna section. Modes 1 and 2 are primarily excited by the multi-branch radiating structure, whereas mode 3 is associated with slot resonance on the CSRRs. For mode 1, the surface currents are strongest along the central branch and weakest at the open end, with the currents on the rectangular ring and the central branch flowing in the same direction. This behavior corresponds to a half-wavelength resonance at 5.1 GHz. For mode 2, two current nulls appear along the rectangular ring, with opposite current directions on either side of each null, corresponding to a full-wavelength resonance at 6.1 GHz. As shown in Figure 4, in comparison to designs without CSRRs, the introduction of the CSRRs not only enhances isolation but also modifies the resonant behavior of the antenna. It can be seen in Figure 5b that this is due to slot-type CSRRs behaving as LC resonators that introduce an additional higher-order resonance, which is a half-wavelength resonance at 6.9 GHz. This newly generated mode merges with the existing patch-based modes, resulting in improved impedance matching and an expanded operating bandwidth. Therefore, the presence of the CSRRs contributes directly to both bandwidth enhancement and the multi-mode behavior of the final design, which reaches 37% and covers the 5.07–7.37 GHz frequency range. Operational requirements of the WiFi 7 high-band can be satisfied.

#### 2.2.2. Analysis of Decoupling Characteristics

**Figure 6:**
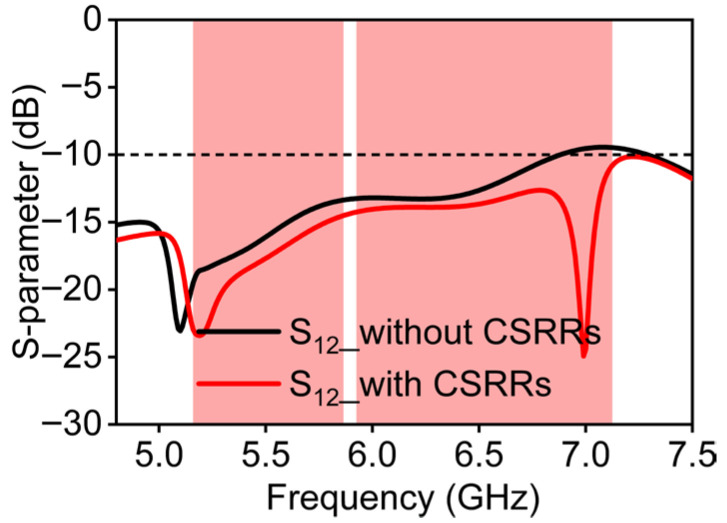
Simulated result comparison of the antenna with and without CSRRs.

If an antenna without CSRRs is used, it can achieve an isolation level above 13.9 dB within the 5 GHz band, as displayed in Figure 6. However, the worst isolation value is 9.8 dB in the 6 GHz band, indicating that the independence between antenna elements is still insufficient. Since the antenna elements share a common copper sheet as a reference ground and the minimum spacing is smaller than half a wavelength, current coupling on the copper ground surface is the primary factor deteriorating the MIMO antenna isolation: the spatial radiation coupling is also noticed. Correspondingly, the decoupling mechanism of the proposed structure is explored in this chapter.

**Figure 7:**
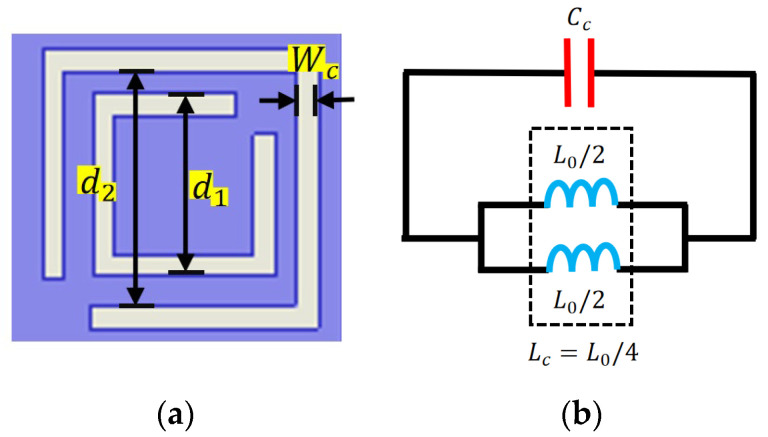
(**a**) CSRR structure. (**b**) Equivalent LC resonate circuit.

According to reference [33], the slots of the CSRRs lie in their ring-slot geometry and act as LC resonators, producing a strong electromagnetic resonance at specific frequencies. The structural parameters of CSRR and its equivalent circuit diagram are shown in Figure 7. When current flows along the CSRR structure, a voltage gradient occurs between the gaps of the CSRR. The ability of the CSRR to interact with electric fields changes the effective dielectric constant near the resonant frequency.

The series capacitance and inductance of the CSRR ring are determined by Equation (1), which is as follows:

(1) $$L_{C}=L_{0}/4$$

$L_{0}$ can be calculated using Equation (2):

(2) $$L_{0}=2\pi d_{m}L_{pul}$$

where $d_{m}$ represents the average length between two rings, and to enhance the rejection band suppression effect the circumferences of the two open rings should be sufficiently close to each other. The $L_{pul}$ represents the inductance per unit length.

$C_{c}$ is the total capacitance of the CSRR, which can be calculated using the following Equation (3):

(3) $$C_{C}=4\frac{\varepsilon }{\mu }L_{s}$$

where ε and μ represent the dielectric constant and magnetic permeability, respectively. And $L_{s}$ can be approximated as an inductance with a single ring with a side length between $d_{1}$ and $d_{1}$ and a width of $W_{c}$.

**Figure 8:**
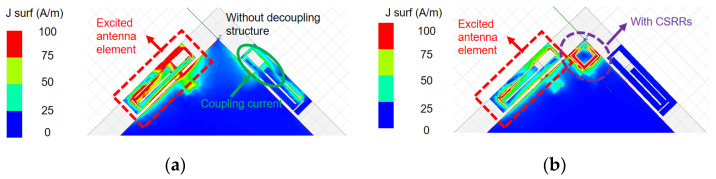
Simulated distributed current results on the aerial surface at 7.0 GHz. (**a**) Single element is excited without a decoupling structure; (**b**) single element is excited with CSRRs.

The simulated current distributions shown in Figure 8 are obtained from excitation of only one antenna port, and matching loads of 50 Ω are applied to the other ports. Analysis focuses on 7.0 GHz, where the isolation performance is relatively poor. As illustrated in Figure 8a, without a decoupling structure the port marked with a red box is excited. The currents can propagate across the copper ground surface and strongly couple into the adjacent non-excited elements. To maintain a compact design and avoid increasing the antenna profile, CSRRs are etched between adjacent antennas.

**Figure 9:**
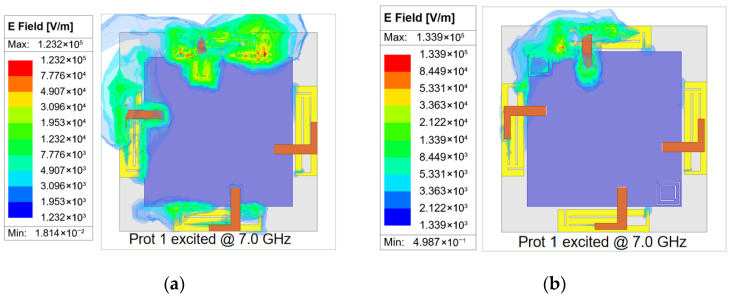
Simulated electric field results at 7.0 GHz. (**a**) Single element is excited without decoupling structure; (**b**) single element is excited with CSRRs.

The resonance of the CSRR introduces a band-stop behavior for surface-wave propagation. This resonance creates a band-stop effect specifically for surface waves, effectively acting as a barrier that prevents their lateral propagation across the ground plane. Specifically, the CSRR geometry can be optimized to confine and resonate surface waves within the resonator, thereby preventing their lateral propagation across the ground plane. Thus, by optimizing the size parameters of the decoupling structures, the surface waves at higher frequencies can be effectively confined and resonated inside the CSRRs. Figure 8b presents the surface current distribution under this condition. When the same port is excited again, the surface currents are directed into the interior of CSRRs and are prevented from coupling to port 2. Therefore, the surface current density on the non-excited element is nearly zero. Moreover, the electric field distribution provides direct evidence of this decoupling mechanism. In Figure 9a, without a CSRR the E-field clearly extends across the ground between adjacent elements, which indicates strong coupling paths. However, in Figure 9b, with a CSRR the E-field concentration inside the rings and the significant reduction between antennas directly demonstrate that the decoupling structures confine surface waves and suppress mutual coupling. Consequently, the proposed antenna achieves an isolation value exceeding 14.5 dB within the 5 GHz band, and a level of over 11.0 dB within the 6 GHz band, as seen in Figure 6. A peak isolation of 25 dB at 7.0 GHz is also observed, providing an effective solution approach for the proposed decoupling design.

**Figure 10:**
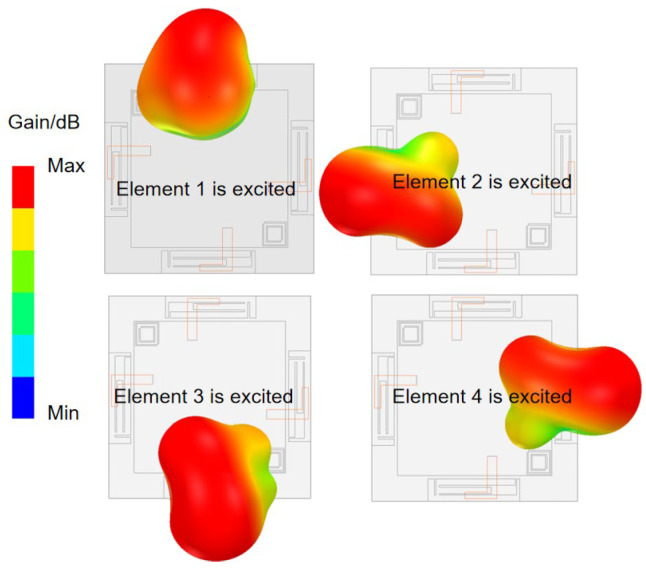
Simulated three-dimensional radiation pattern of different MIMO elements.

In addition to suppressing current coupling on the copper surface, spatial radiation coupling can also degrade port isolation. In this work, radiation diversity is achieved by arranging the four antenna elements in orthogonal directions, ensuring that the radiated electric fields from one element do not excite resonances in the others. Figure 10 illustrates the simulated three-dimensional radiation pattern. The directions of the adjacent elements’ radiation pattern are approximately perpendicular. This configuration effectively minimizes the spatial correlation between the radiation pattern, thereby further reducing mutual interference among the MIMO antenna elements. However, this approach also presents limitations. The achievable isolation improvement decreases at lower frequencies, where the element spacing becomes electrically small. And orthogonal placement may restrict layout flexibility in highly integrated router platforms.

#### 2.2.3. Parametric Analysis

**Figure 11:**
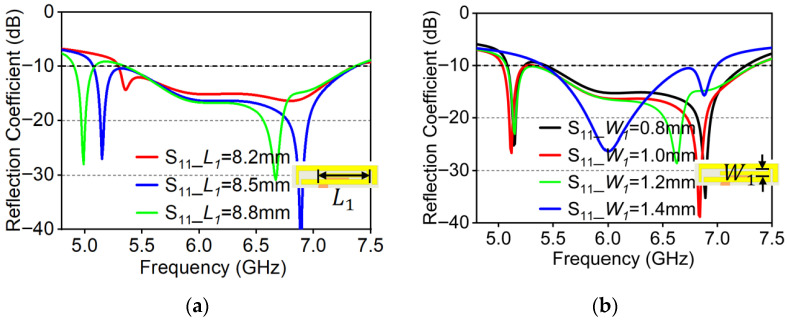
Simulated reflection coefficient under parameters variations. (**a**) Variation of $L_{1}$; (**b**) variation of $W_{1}$.

As the branch length ($L_{1}$) increases, the operation range shifts toward the lower frequency band and the impedance bandwidth becomes narrower. It primarily affects the first resonant mode around 5.2 GHz, as shown in Figure 11a.

This phenomenon occurs because the low-frequency mode is concentrated in the central part of the radiation patches. Increasing the branch length enhances its inductance, thereby extending the resonant current path and shifting the resonant frequency downward. Meanwhile, the increased distributed inductance also raises the Q-factor of this mode, resulting in a narrower bandwidth. Considering the requirement of covering the WiFi operating band for practical applications, $L_{1}=8.5\;mm$ is selected as the optimal branch length.

As shown in Figure 11b, within a certain range the branch width ($W_{1}$) significantly influences the second resonant mode, because this mode is mainly concentrated in the rectangular ring slot region. As $W_{1}$ widens, the slot gradually becomes narrower, leading to a larger distributed capacitance and a downward shift in resonant frequency. However, when $W_{1}\geq 1.4\;mm$, the low-frequency resonant mode is destroyed. Considering impedance matching, $W_{1}=1.0\;mm$ is adopted as the optimal width for this structure.

**Figure 12:**
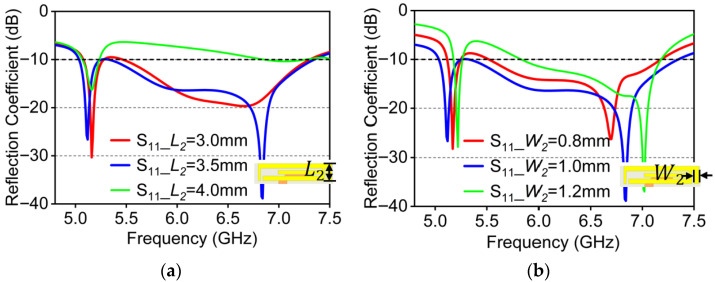
Simulated reflection coefficient under parameters variations. (**a**) Variation of $L_{2}$; (**b**) variation of $W_{2}$.

As depicted in Figure 12a, $L_{2}$ is the bent branch length. Along with it decreasing within a certain range, the resonant modes of the antenna tend to merge toward the mid-frequency region, resulting in a reduced impedance bandwidth. When $L_{2}\geq 4.0\;mm$, the branch is directly connected to the copper sheet, causing a short-circuit path that severely disturbs the resonance and causes significant impedance mismatch at higher frequencies. Considering reflection performance and fabrication feasibility, $L_{2}=3.5\;mm$ is chosen as the bent branch length.

Figure 12b displays the influence of width of the bent branch ($W_{2}$) for the antenna reflection coefficient variation. The influence of parameter $W_{2}$ is significant for overall antenna impedance. As $W_{2}$ widens, the resonant frequency shifts toward the left. When $W_{2}=1.0\;mm$, the antenna achieves better impedance matching. That width is larger or smaller, which can deteriorate the impedance matching performance and prevent the antenna from effectively covering the targeted WiFi band.

## 3. Implementation and Performance of the 4×4 MIMO Antenna Prototype

**Figure 13:**
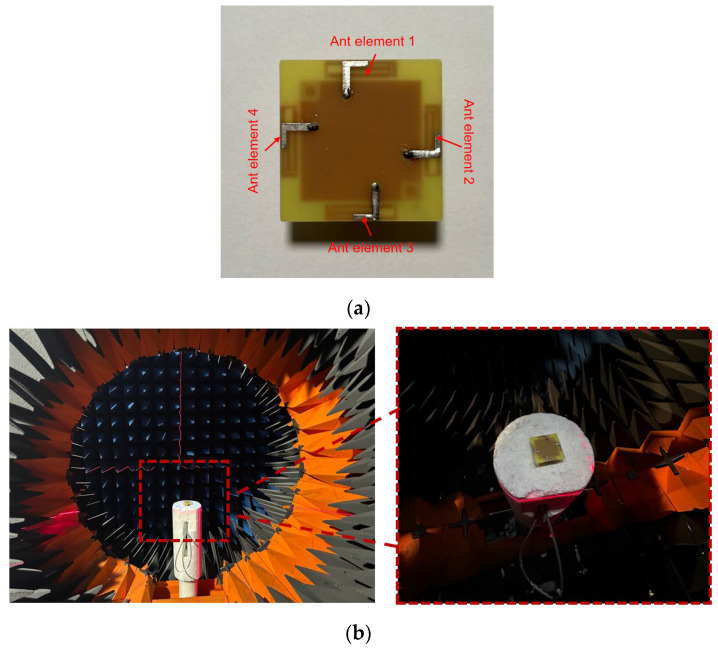
MIMO antenna fabrication prototype display. (**a**) Top sight; (**b**) measurement environment.

Pictures of the four-port MIMO antenna fabrication prototype can be observed via Figure 13. Four 50 Ω SMA connectors are employed on the bottom side of a PCB for measurement. The antenna is measured using an E5071C vector network analyzer and an 85052D calibration kit (both from Keysight Technologies, Santa Rosa, CA, USA). The measured environment is displayed in Figure 13b, which is the MVG spherical near-field anechoic chamber. And far-field results are calculated based on near-field measured results.

### 3.1. Measured Results of 4 × 4 MIMO Antenna Prototype

**Figure 14:**
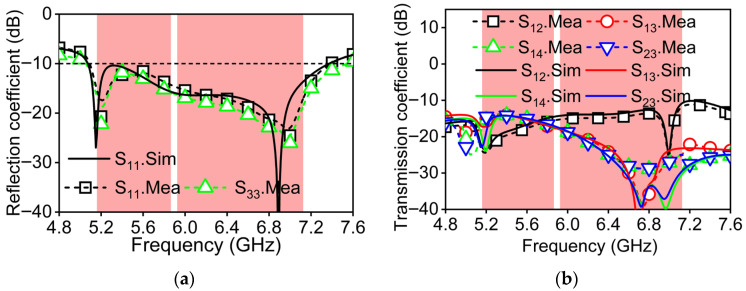
Simulated and measured S-parameter of 4 × 4 MIMO antenna prototype. (**a**) Reflection coefficient; (**b**) transmission coefficient.

The reflection coefficients and isolation characteristics of the proposed MIMO antenna, both simulated and measured, are shown in Figure 14. Compared with the simulation results, there is a phenomenon of the test results shifting towards higher frequencies. Because the CSRR resonance is highly sensitive to the slot width and ring width, typical PCB fabrication tolerances may cause a shift in the resonant frequency. In Figure 14a, the measured antenna reflection coefficient result indicates good impedance matching, achieving a reflection coefficient below −10 dB across both the aimed WiFi bands, which are 5.150–5.835 GHz and 5.925–7.125 GHz. In the 5 GHz band, poor isolation occurs between element 1 and element 3. Differently, in the 6 GHz band, poor isolation occurs between element 1 and element 2. This difference is due to the variation in electric field coupling caused by multi-resonant modes of the antenna. The measured isolation value within the 5 GHz band exceeds 14.5 dB. In the 6 GHz band, the measured isolation results remain above 11.5 dB and reach a maximum of 20 dB, fulfilling the isolation requirements for MIMO terminal devices such as wireless routers.

**Figure 15:**
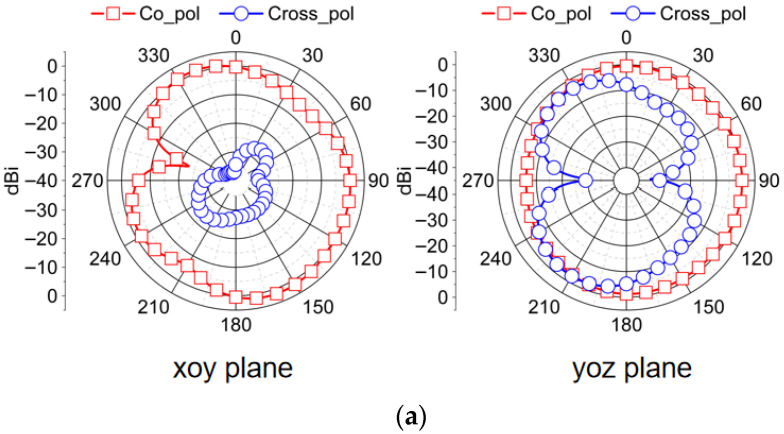

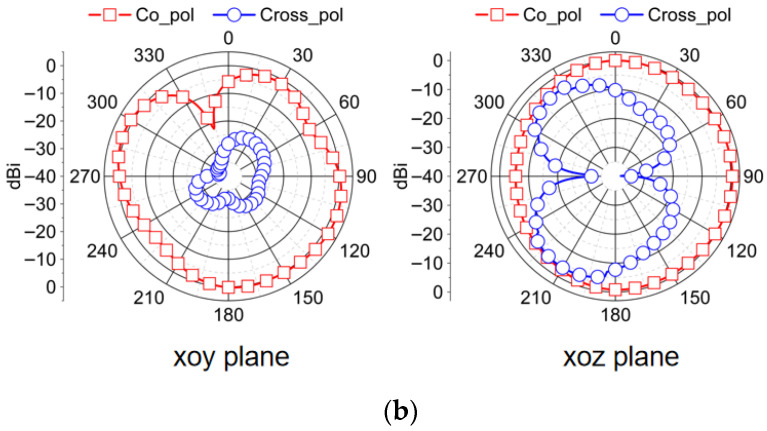
Radiation pattern obtained by measuring the proposed MIMO antenna at 5.6 GHz. (**a**) When antenna 1 is excited; (**b**) when antenna 2 is excited.

**Figure 16:**
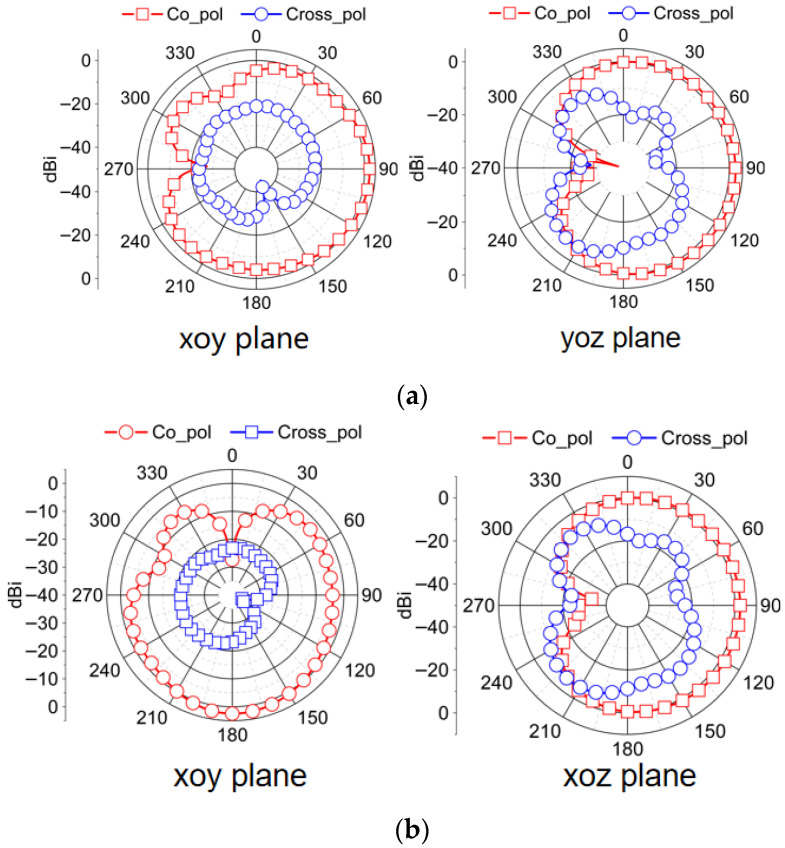
Radiation pattern obtained by measuring the proposed MIMO antenna at 6.5 GHz. (**a**) When antenna 1 is excited; (**b**) when antenna 2 is excited.

The two-dimensional radiation pattern of the designed antenna in the horizontal plane (xoy plane) and the vertical planes (yoz or xoz planes) is observed when antenna 1 and antenna 2 are excited, respectively. When one port is stimulated, other ports are connected to a 50 ohm load. Figure 15 is the radiation pattern at 5.6 GHz, and Figure 16 is the radiation pattern at 6.5 GHz. It can be seen in both bands that the radiation patterns from the co-polarization of different antenna elements align along distinct directions. When the frequency increases, the diversity of the antenna elements becomes significant. And in a plane parallel to the ground, the radiation pattern of cross-polarization is significantly lower than the radiation pattern of co-polarization, achieving high polarization purity because edge effects have negligible influence in this plane. Conversely, in a plane perpendicular to the ground, the deterioration of polarization purity is due to the enhancement of edge effects. In conclusion, the maximum radiation direction of elements along different angles increases the isolation between the ports and introduces polarization diversity. This radiation direction diversity due to layout optimization also ensures that MIMO antennas present no radiation blind spots, exhibiting approximate omnidirectional characteristics.

**Figure 17:**
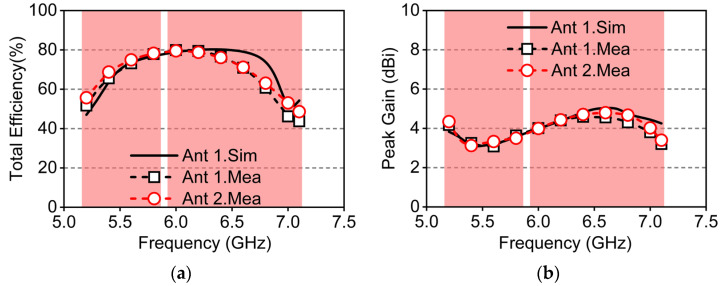
Simulated and measured total efficiency and peak gain of the proposed MIMO antenna. (**a**) Total Efficiency; (**b**) peak gain.

Figure 17 presents the total efficiency and peak gain of the designed four-port MIMO antenna. Within the 5 GHz band, the measured efficiency exceeds 50%, reaching a maximum of 79%. In the 6 GHz band, the efficiency remains above 45%, with a maximum of 80%. Across the target frequency bands, the peak measured gain value for the antenna is in excess of 3.0 dBi, satisfying the design requirements for MIMO antennas in wireless routers. It can be seen that compared with the 5G band, there is a significant variation between the measured and simulated values of the antenna’s total efficiency and gain in the 6 GHz band. It can be inferred that this is due to higher operation frequency, connector insertion loss, and increased dielectric substrate loss.

### 3.2. Diversity Performance of 4 × 4 MIMO Antenna Prototype

**Figure 18:**
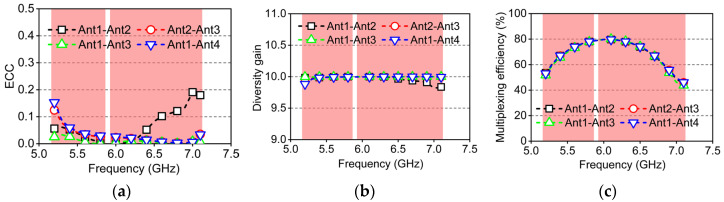
Measured diversity and multiplexing performance of the proposed MIMO antenna. (**a**) ECC; (**b**) diversity gain; (**c**) multiplexing efficiency.

For the MIMO system to enhance the transmission rate, it relies on spatial multiplexing and diversity techniques. Therefore, the diversity and multiplexing characteristics of the antenna are crucial and need to be discussed further. The ECC is used to evaluate the correlation between multiple antenna elements and is usually calculated by the scattering coefficient or the radiation pattern of the antenna. The ECC values are calculated from the S-parameter measured data from ports m and n using Equation (4). The calculation results are plotted in Figure 18a. The ECC remains below 0.16 in the 5G band and below 0.2 in the 6G band, satisfying the spatial correlation requirements for terminal MIMO antennas (<0.5).

(4) $$ECC(m,n)=\frac{{\left|S_{mm}^{*}S_{mn}+S_{nm}^{*}S_{nn}\right|}^{2}}{\left\{1-({\left|S_{mm}\right|}^{2}+{\left|S_{nm}\right|}^{2})\}\{1-({\left|S_{nn}\right|}^{2}+{\left|S_{mn}\right|}^{2})\right\}}$$

The DG is an indicator that measures the ability of an antenna system to resist signal fading and is used to evaluate the diversity performance of MIMO antennas. Specifically, diversity technology reduces signal fading caused by a single path by transmitting information through multiple antenna units and utilizing multiple independent channels. Ideally, DG should be as close as possible to 10 dB to ensure the reliability of the system’s ability to resist signal fading. The DG can be calculated from the ECC values using Equation (5). It can be obtained from Figure 18b that the DG of the 4 × 4 MIMO antenna approaches 10.

(5) $$DG(m,n)=10\sqrt{1-{ECC(m,n)}^{2}}$$

The port multiplexing efficiency of the proposed MIMO antenna can be calculated using Equation (6), where $\eta_{m}$ and $\eta_{n}$ represent the total efficiencies of ports m and n, respectively, and $\rho_{mn}$ is the correlation coefficient between the two ports. As shown in Figure 18c, the multiplexing efficiency ranges from 45% to 80%, further demonstrating that the proposed MIMO antenna exhibits good diversity and multiplexing performance.

(6) $$\eta_{mux}\left(m,n\right)=\sqrt{\eta_{m}\eta_{n}(1-\left|{\rho_{mn}}^{2}\right|)}$$

## 4. Conclusions

This work presents a miniaturized 4×4 MIMO antenna specifically to be used with WiFi 7 systems. By integrating multi-branch radiating patches with coupled microstrip feeds, the antenna realizes multi-mode operation, thereby achieving an impedance bandwidth of 37%, which fully encompasses the WiFi 7 high-frequency bands. To mitigate inter-element coupling while maintaining a compact profile, two CSRRs are introduced between MIMO elements to suppress surface current interaction, and the orthogonal configuration of the elements further improves isolation. The fabricated prototype exhibits good consistency between simulations and measurements, attaining isolation above 11 dB in the working band. Moreover, the antenna maintains high radiation efficiency, a low ECC, and favorable diversity performance. Although the proposed antenna demonstrates excellent isolation and diversity characteristics in controlled measurement conditions, its performance in real-world multi-user and multi-path environments has not yet been experimentally evaluated. In practical WLAN scenarios, dynamic user movement, device orientation, and interference from nearby access points may introduce additional correlation and link degradation. Future work will therefore include over-the-air (OTA) testing in a channel-emulation environment and full MIMO throughput measurements to further assess system-level performance. Although the proposed antenna demonstrates excellent isolation and diversity characteristics in controlled measurement conditions, in practical WLAN scenarios, dynamic user movement, device orientation, and interference from nearby access points may introduce additional correlation and link degradation. Future work can take over-the-air (OTA) testing in a channel-emulation environment and MIMO throughput measurements to further assess system-level performance. Overall, the proposed design provides a practical, robust, and efficient MIMO antenna solution suited for WiFi 7 devices such as wireless access points and routers.

## Data Availability

The original contributions presented in this study are included in the article. Further inquiries can be directed to the corresponding author.

## Abbreviations

The following abbreviations are used in this manuscript:

MIMO	Multiple-input multiple-output
CSRRs	Complementary split-ring resonators
ECC	Envelope correlation coefficient
DG	Diversity gain
SRR	Split-ring resonators
SIR	Step-impedance resonator
OTA	Over-the-air
